# Non-pharmacological intervention for rehabilitation of post-stroke spasticity: A protocol for systematic review and network meta-analysis

**DOI:** 10.1097/MD.0000000000025788

**Published:** 2021-05-07

**Authors:** Guanyu Hu, Hongshi Zhang, Yufeng Wang, Deyu Cong

**Affiliations:** Changchun University of Chinese Medicine, Changchun, China.

**Keywords:** network meta-analysis, rehabilitation, spasticity, stroke, systematic review

## Abstract

**Background:**

Post-stroke spasticity (PSS) is a major worldwide health problem, and timely and effective rehabilitation is associated with the risk of diabetes development; there are a variety of non-pharmacological interventions applied to the rehabilitation of PSS in these treatments; however, the relative efficacy and safety of different therapies remain uncertain, and we will conduct a systematic review and network meta-analysis to evaluate different non-pharmacological interventions. The relative efficacy and safety of intervention in PSS rehabilitation, thus providing evidence to support the optimization of the PSS rehabilitation program.

**Methods:**

We searched the following databases electronically, including four English literature databases (i.e., PubMed, Medline, Embase, and Cochrane Library) and two Chinese literature databases (i.e., China National Knowledge Infrastructure and VIP). We will also search for randomized controlled trials on non-pharmacological interventions for post-stroke spasticity, and the search time limit is from its establishment to May 2020. Two reviewers working independently will screen the titles, abstracts, and full papers. Data extraction will be completed by two independent authors. The primary outcome was the motor function. The secondary outcome was the assessment of daily living ability. We will use RevMan V.5.3 software to compute the data synthesis carefully when a meta-analysis is allowed. We will conduct Bayesian network meta-analysis using the Markov Chain Monte Carlo random effects model in Aggregate Data Drug Information System version 1.16.8 (Drugis, Groningen, NL).

**Results:**

This study provides a high-quality synthesis to assess the effectiveness and safety of non-pharmacological interventions for patients with PSS.

**Conclusion:**

The results of this study will provide evidence to judge whether non-pharmacological interventions are effective interventions for patients with post-stroke spasticity.

**Ethics and dissemination:**

The results of this meta-analysis and meta-regression will be disseminated through publication in a peer-reviewed journal and presented at a relevant conference. The data used in the network meta-analysis did not contain individual patient data. Therefore, ethical approval was not required.

**INPLASY registration number:**

INPLASY202140059

## 1 Introduction

Stroke is characterized by high morbidity, mortality, and disability.^[1]^ Post-stroke spasticity(PSS) is the most common complication of stroke. It is estimated that approximately 20% to 40% of stroke survivors will have limb spasms.^[2]^ Research on the occurrence time and degree of PSS showed that the incidence of spasm within 1 month after stroke was 42.6%, of which severe spasm was 15.6%.^[3]^ The incidence of spasms at 3 months is approximately 19%.^[4]^ The incidence of spasms within 6 months in stroke patients is 21.7% to 23%.^[5,6]^ PSS seriously affects the motor function of patients, and therefore seriously affects the living standards and prognosis of patients.^[7]^ Therefore, it is very important to select a safe and cost-effective PSS treatment and rehabilitation method. Treatments of PSS include oral anti-spastic medication, BTX-A injections, surgical interventions, and physiotherapy or a combination of the aforementioned therapies. A study has shown that medication can relieve spasms caused by central nerve injury and may also cause muscle weakness.^[8]^ In clinical treatments, physiotherapy is a rehabilitation intervention for PSS, such as Bobath, which is widely used in the treatment of PSS.^[9]^ To improve the rehabilitation efficiency of PSS, most medical institutions combine a variety of characteristic non-pharmacological therapies based on conventional rehabilitation therapies for PSS. Hospitals of traditional Chinese medicine (or rehabilitation departments of traditional Chinese medicine) often combine electric acupuncture, Tui-na, medicinal bath, and other therapies in the rehabilitation of PSS.^[10]^ It is difficult to optimize the clinical rehabilitation program of PSS because of the wide variety of non-pharmacological therapies that have rehabilitative effects on PSS. Therefore, it is of great significance for the rehabilitation of PSS to select intervention methods with higher cost performance among various interventions.

Network meta-analysis enables the comparison of multiple interventions to incorporate clinical evidence for direct and indirect treatment comparisons in treatment and related trial networks.^[11]^ To date, this is the first time that network meta-analysis has been used to compare currently available methods for multiple interventions to determine the effectiveness of non-pharmacological interventions in the PSS.

## 2 Methods

The Bayesian network meta-analysis (NMA) method was adopted in the present work, following the PRISMA-P guidelines.

### 2.1 Study registration

The present NMA was registered at International Platform of Registered Systematic Review and Meta-analysis Protocols (INPLASY registration number: INPLASY202140059. Registration No.: URL= https://inplasy.com/inplasy-2021-4-0059/. DOI number:10.37766/inplasy2021.4.0059).

### 2.2 Inclusion criteria for study selection

#### 2.2.1 Types of studies

All clinical randomized controlled trials (RCTs) of non-pharmacological interventions for rehabilitation of PSS will be included in the review.

#### 2.2.2 Participants

The cases included in the trial were all patients with PSS (diagnosed using any recognized diagnostic criteria), not limited by age and race.

#### 2.2.3 Interventions

The treatment group adopted non-pharmacological intervention (i.e., acupuncture, dry acupuncture, Tui-na, medicated bath, music therapy, etc., without restricting the choice of operation method and course of treatment), while the control group adopted internationally recognized treatment methods or routine treatment(such as rehabilitation training).

#### 2.2.4 Outcomes

##### 2.2.4.1 Primary outcomes

The primary outcome indicator was the assessment of motor function, and the included RCTs included at least one of the Modified Ashworth Scale and the Fugl-Meyer Assessment.

##### 2.2.4.2 Secondary outcomes

Secondary outcome indicators were daily living ability assessment, including the Barthel index rating scale and daily living ability scale.

### 2.3 Database and search strategy

#### 2.3.1 Electronic searches

The following databases will be searched electronically, including four English literature databases (i.e., PubMed, Embase, MEDLINE, and Cochrane Library) and two Chinese literature databases (i.e., China National Knowledge Infrastructure and VIP). We will also search for RCTs on non-pharmacological interventions for post-stroke spasticity, and the search time limit is from its establishment to April 2021. In addition, we will retrieve unpublished protocols and summarize the results by searching the clinical trial registry at https://ClinicalTrials.gov. The search used a combination of subject words and free words, and the search strategy was determined after multiple presearches. The search terms included post-stroke, spastic hemiplegia, spasticity, treatment, intervention, and randomization. Meanwhile, we will search the literature included in the research reference and original literature, which are subject related and included in systematic reviews, to supplement and obtain relevant literature and ensure the recall ratio. The detailed search strategy is presented in Table 1.

**Table 1:** Detailed search strategy in PubMed.

No.	Search Item
#1	“post-stroke”[Title] OR “after stroke”[Title]
#2	“spastic hemiplegia”[Title] OR “spasticity”[Title] OR “Limb spasm”[Title]
#3	“spasticity after stroke”[Title] OR “post-stroke spasticity”[Title] OR “post-stroke hemiplegia”[Title] OR “post-strokespastic hemiplegia”[Title]
#4	#1 AND #2
#5	“treatment”[Abstract] OR “intervention”[Abstract] OR “therapy”[Abstract] OR “management” [Abstract] OR “rehabilitation” [Abstract]
#6	“controlled clinical trial”[Title] OR “randomized controlled trail”[Title] OR “randomized”[Title] OR “placebo or randomly”[Title] OR“ trial or groups”[Title]
#7	#3 OR #4
#8	#5 AND #6 AND #7

#### 2.3.2 Searching other resources

We will also manually retrieve relevant conference reports and contact experts in the field and corresponding authors to obtain important information that cannot be obtained by the above retrieval.

### 2.4 Study selection and data extraction

#### 2.4.1 Selection of studies

**Figure 1:**
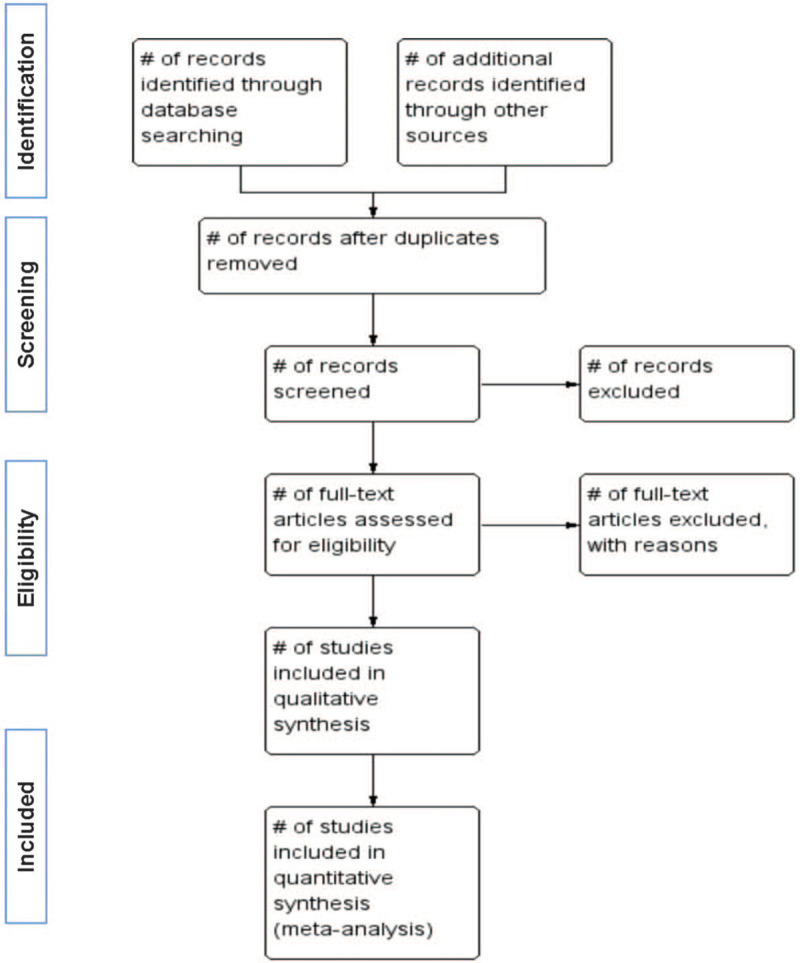
Flow diagram of study selection process.

Researchers will discuss and determine the screening criteria within the group before searching for studies. The corresponding research members will import the retrieved studies into the document management system of EndnoteX7 for repetition removal. We will then exclude the apparently unqualified literature by reading the headings and abstracts, and determine the final included literature by reading the full text, discussing within the group, and contacting the author to know more about the research details. The final list of included studies will be converted into Microsoft Excel. Both information retrieval and literature screening will be independently performed by two research members. Finally, another research member will resolve the inconsistency and check the final included studies. The study selection is summarized in the PRISMA flow diagram (Fig. 1).

#### 2.4.2 Data extraction and management

Two independent reviewers extracted data from selected studies using pilot-tested data forms. They will include the following information: author, year of publication, study population, study design, number of patients randomized and treated, number of patients analyzed, baseline analysis, random sequence generation, allocation concealment method, blinding method, imputation method, withdrawals of data, interventions, controls, medication records, and primary and secondary outcomes at all reported time points. To investigate the characteristics of non-pharmacological intervention effects, we extracted data on age, sex, population, number and duration of treatment sessions, features of non-pharmacological intervention (such as frequency of stimulation and point of treatment), features of control interventions (sham methods or standard treatment details), and patient expectations. We also document for each outcome of the percentage of missing values reported in the study, and any disagreement on data collection will be resolved through discussions or negotiations with the third arbitrator. If the data provided in the study are unclear, missing, or presented in a form that is not extractable or difficult to extract reliably, we will contact the author of the study for clarification.

### 2.5 Risk of bias assessment

According to the Cochrane Collaboration's tool for assessing risk of bias provided by the Cochrane Handbook for Systematic Reviews of Interventions, seven dimensions will be assessed from 7 dimensions: random sequence generation, allocation concealment, blinding of patients, blinding of testers, blinding of outcome evaluators, outcome data incompletion, and selective reporting of seven dimensions for evaluation. The results of the assessment were divided into three levels: low risk, unclear, and high risk. The assessment will be conducted independently by two trained research members, and the inconsistencies will be resolved through intragroup discussions, contacting authors to determine details with the third-party arbitrator.

### 2.6 Measures of treatment effect

The enumeration data were expressed as relative risk, the measurement data adopted mean difference, and each effect amount was expressed in a 95% confidence interval.

### 2.7 Dealing with missing data

For studies in which the data were missing, the researcher tried to obtain information by contacting the corresponding author of the study. If contact is lost, we build our analysis on the available data.

### 2.8 Assessment of heterogeneity

Meta-analysis will be performed using RevMan 5.3 software. When there was no statistical heterogeneity among the results, a fixed-effects model was used for the meta-analysis. When there was statistical heterogeneity among the results, the heterogeneity source was further analyzed, and a random-effects model was used for meta-analysis after excluding the effects of significant clinical heterogeneity. When there is significant clinical heterogeneity, we will use subgroup analysis, sensitivity analysis, or only descriptive analysis.

### 2.9 Assessment of reporting bias

Reporting bias will be explored by constructing funnel plots and performing Egger test if there were at least 10 trials included in the meta-analysis.

### 2.10 Data synthesis

The synthesis will be performed by generating a forest plot for meta-regression. This plot does not contain a summary measure given by a prism below the single studies, but by a prism shown for each single study that shows the aggregated effect for the specific type of study (depending on the covariates of the meta-regression). If the heterogeneity test indicated that there was no substantial heterogeneity between studies, the Mantel-Haenszel method was fitted to calculate pooled estimates, 95% CIs, and combined *P* values. If substantial heterogeneity is indicated by *I*^2^ 50%, the random-effects model will be performed using the DerSimonian and Laird method (DerSimonian 1986) and the rma function. The significance of the *P*-value represents the strength of evidence against the null hypothesis of no intervention effect. We will conduct Bayesian NMA using the Markov Chain Monte Carlo random effects model in Aggregate Data Drug Information System version 1.16.8 (Drugis, Groningen, NL). We will network the translated outcomes within studies and specify the relations among the MD across studies, making different comparisons, as previously reported. We used *P* < .05, and 95% CI beyond the null value to assess significance. We also calculated the inconsistency factor (IF) and 95% CI to evaluate the inconsistency of each closed loop, with an IF close to 0. In addition, the random effects variance and inconsistency variance were roughly equal, which is considered to be less inconsistent.

### 2.11 Subgroup analysis

The following subgroup analysis will be performed to assess the heterogeneity of the research:

(1) Different types of non-pharmacological interventions.
(2) Different intervention times for Windows
(3) Upper or lower limbs.
(4) Different regions of the study

In addition, if we detect any important and significant covariate contributing to the variation of the intervention effect by meta-regression, subgroup analyses will also be conducted according to these covariates.

### 2.12 Sensitivity analysis

To confirm the robustness of our findings, a sensitivity analysis was conducted based on the different levels of bias of the included studies. To evaluate the internal validity of studies or treatment adequacy, we will subsequently remove studies with a high risk of bias, studies of unclear risk of bias, and studies of low risk of bias using the meta for package and leave1out function.

### 2.13 Summary of evidence

We will summarize the quality of evidence using the Grading of Recommendations Assessment, Development and Evaluation (GRADE) approach38 and present a summary of findings tables. The summary of findings tables will be generated by the GRADE working group software (GRADEpro or GRADEpro GDT (www.gradepro.org). The summary of findings tables (main outcomes that are important to patients and decision makers) will be determined by the review group described above. Where possible, both relative and absolute measures of the effect will be provided. To assess the quality of evidence, the GRADE approach evaluates the quality of evidence as high, moderate, low, or very low based on the outcome. Evidence can be downgraded in category by concerns about risk of bias, imprecision, inconsistency, indirectness, or publication bias, and can also be upgraded by a large effect size, which could change the effect size and dose response relation. Reviewers will downgrade or upgrade the evidence according to the GRADE guidelines in the Cochrane handbook, Chapter 1134, and also take into account the differences in anticipated effects in the group of primary interest. The total quality of the evidence will be adjudicated on the basis of both reviewers and all members of the review board.

### 2.14 Ethics and dissemination.

Since confidential patient data will not be included in this study, formal ethics approval is not required. The framework of the PRISMA statements for NMA will be applied to guide the review authors to perform this study. In addition, the findings will be disseminated through conference presentations and peer-reviewed publications.

## 3 Discussion

The occurrence of PSS is related to the damage of upper motor neurons after stroke,^[12]^ and its pathogenesis is complex, and is a type of motor dysfunction that often occurs after a stroke. This type of motor dysfunction is indicated by a velocity-dependent increase in tonic stretch reflexes due to hyperexcitability of the stretch reflex. Studies have shown that non-drug therapies such as acupuncture, rehabilitation training, and physical therapy can reduce limb spasms and promote the recovery of upper motor neurons.^[13,14]^ Therefore, in the treatment of PSS, in addition to the basic treatment of stroke and oral anti-spastic medicine therapy, it is necessary to promote the rehabilitation effect of PSS by combining the application of non-pharmacological therapy such as exercise therapy, rehabilitation training, and physical therapy. However, in the rehabilitation of PSS, the choice of non-pharmacological therapy is still lacking scientific evidence.

This research is a comprehensive systematic review and NMA to compare various non-pharmacological interventions for the treatment of PSS. Our study will provide clinicians and guideline makers with available evidence on various non-pharmacological interventions for the prevention and treatment of PSS. At present, there is no NMA for the prevention and treatment of PSS using non-pharmacological interventions. In addition, different hospitals adopt different non-pharmacological interventions for the prevention and treatment of PSS, which lacks a more standard and optimized clinical implementation program. Therefore, we plan to carry out this study, and our findings will lead to high-quality recommendations on the best external Chinese medicine treatment for the prevention and treatment of PSS, and provide evidence for the optimization of prevention, treatment, and rehabilitation programs for PSS. However, our study has several limitations. First, due to the difficulty in quantifying some non-pharmacological interventions, there are differences in the actual operation of homeopathy in different studies, resulting in considerable heterogeneity. Second, differences between the inclusion criteria of participants and the definition of primary endpoint events may affect the quality of evidence. Third, due to the innate characteristics of some therapies, it is difficult to achieve double blindness, which may affect the quality of evidence. Finally, research-level data will be used, rather than individual data. If this protocol must be amended, we will present the date of each amendment with a description of the change and the corresponding rationale.

## Author contributions

Deyu Cong is the guarantor of this article. The manuscript was drafted by Deyu Cong and Guanyu Hu. Hongshi Zhang and Yufeng Wang developed a search strategy. Guanyu Hu and Hongshi Zhang independently screened the potential studies and extracted the data. Guanyu Hu and Deyu Cong assessed the risk of bias and finished data synthesis. Deyu Cong arbitrated any disagreement and ensured that no errors occurred during the review. All review authors critically reviewed, revised, and approved the final version of the protocol.

**Conceptualization:** Deyu Cong.

**Data curation:** Guanyu Hu.

**Formal analysis:** Guanyu Hu.

**Funding acquisition:** Yufeng Wang.

**Methodology:** Hongshi Zhang, Yufeng Wang.

**Software:** Guanyu Hu.

**Writing – original draft:** Guanyu Hu.

**Writing – review & editing:** Guanyu Hu.
